# STABLE trial of spectacle provision and driving safety among myopic motorcycle users in Vietnam: study protocol for a stepped-wedge, cluster randomised trial

**DOI:** 10.1186/s13063-024-08644-2

**Published:** 2024-12-18

**Authors:** Vinh Chi Le, Kien Gia To, Van Dat Le, Le Nguyen, Graeme MacKenzie, Lovemore Nyasha Sigwadhi, Prabhath Piyasena, Mai Tran, Ving Fai Chan, Rohit C. Khanna, Mike Clarke, Lynne Lohfeld, Heather Dickey, Augusto Azuara-Blanco, Asha Latha Mettla, Sridevi Rayasam, Han Thi Ngoc Doan, Dung Van Do, Phuoc Hong Le, Charlie Klauer, Richard Hanowski, Zeb Bowden, Lynn Murphy, Joanne Thompson, Susan McMullan, Cliona McDowell, Raja Narayanan, Julie-Anne Little, Huong Thu Ha, Sangchul Yoon, Rahul Goel, Lan Luong, Xuan Nguyen, Nathan Congdon

**Affiliations:** 1 Centre for Public Health, School of Medicine Dentistry and Biomedical Sciences, Queen’s University Belfast, Belfast, BT12 6BA UK; 2 Faculty of Public Health, University of Medicine and Pharmacy at Ho Chi Minh City, 217 Hong Bang Street, District 5, Ho Chi Minh City, Vietnam; 3 Transport Development and Strategy Institute, Hanoi, Vietnam; 4 Asia Injury Prevention Foundation, Hanoi, Vietnam; 5 Riemann Limited, London, UK; 6 Hyderabad Eye Research Foundation, L V Prasad Eye Institute, Hyderabad, Telangana India; 7 Vision and Eye Research Institute, Anglia Ruskin University, Cambridge, UK; 8 Allen Foster Community Eye Health Research Centre, Gullapalli Pratibha Rao International Centre for Advancement of Rural Eye Care, L V Prasad Eye Institute, Hyderabad, India; 9 Brien Holden Eye Research Centre, L.V. Prasad Eye Institute, Banjara Hills, Hyderabad, India; 10 School of Optometry and Vision Science, University of New South Wales, Sydney, Australia; 11 School of Medicine and Dentistry, University of Rochester, Rochester, NY USA; 12 Northern Ireland Clinical Trials Unit, Belfast, UK; 13 Centre for Health Research at the Management School Economics, Queen’s University Belfast, Belfast, UK; 14 Virginia Tech Transportation Institute, Blacksburg, USA; 15 Centre of Optometry and Vision Science, School of Biomedical Sciences, Ulster University, Coleraine, UK; 16 Hanoi Medical University, Hanoi, Vietnam; 17 Department of Medical Humanities and Social Sciences, Yonsei University College of Medicine, Seoul, Republic of Korea; 18 Transportation Research and Injury Prevention Centre, Indian Institute of Technology, Delhi, New Delhi, India; 19 Eye Care Foundation, Ho Chi Minh, Vietnam; 20 Department of Preventive Ophthalmology, Zhongshan Ophthalmic Center, Guangzhou, Guangdong China; 21 Orbis International, New York, USA

**Keywords:** Road traffic crash, Naturalistic driving, Myopia, Motorcycle, Stepped-wedge randomised trial, Vietnam

## Abstract

**Background:**

Traffic crashes are the leading cause of death globally for people aged 5–29 years, with 90% of mortality occurring in low- and middle-income countries (LMICs). The STABLE (Slashing Two-wheeled Accidents by Leveraging Eyecare) trial was designed to determine whether providing spectacles could reduce risk among young myopic motorcycle users in Vietnam.

**Methods:**

This investigator-masked, stepped-wedge, cluster randomised naturalistic driving trial will recruit 625 students aged 18–23 years, driving ≥ 50 km/week, with ≥ 1-year driving experience and using motorcycles as their primary means of transport, in 25 clusters of 25 students in Ho Chi Minh City, Vietnam. Motorcycles of consenting students who have failed self-testing on the WHOeyes app will be fitted with Data Acquisition Systems (DAS) with video cameras and accelerometers. Video clips (± 30 s) of events flagged by the accelerometer will be reviewed for crash and near-crash events per 1000 km driven (main outcome). Five clusters of 25 students will be randomly selected every 12 weeks to undergo ocular examination and an estimated 40% of these will have bilateral spherical equivalent < − 0.5 D, and better-eye presenting distance visual acuity < 6/12, correctable bilaterally to ≥ 6/7.5. They will be given free distance spectacles and their driving data before receiving spectacles will be analysed as the control condition and subsequent data as the intervention condition. Secondary outcomes include visual function, cost-effectiveness and self-reported crash events.

**Discussion:**

STABLE will be the first randomised trial of vision interventions and driving safety in a LMIC.

**Trial registration:**

ClinicalTrials.gov, NCT05466955. Initial registration: 20 July 2022, most recent update: 9 July 2024.

## Introduction

Road traffic crashes are the leading cause of death globally among people aged 5 to 29 years and are predicted to become the seventh most-common cause of all-age mortality by 2030 [1, 2]. Though only 60% of the world’s cars are driven in low- and middle-income countries (LMICs), 90% of traffic crash mortality occurs there [1]. Southeast Asia trails only Africa as the global region with the highest burden of traffic mortality, with 20.7 fatalities per 100,000 population [3]. Drivers aged < 25 years are 15 to 33 times more likely to crash than older drivers [4]. More than 80% of Vietnamese households own at least one motorcycle, which comprise 93% of registered motor vehicles in the country [5]. Southeast Asia is the global region with the highest number of deaths associated with motorcycle use, accounting for 43% of global fatalities, with Vietnam, where motorcycles are involved in over two-thirds of road fatalities, having one of the region’s highest rates of motorcycle-related mortality [3].

Myopic refractive error is the leading cause of distance vision impairment globally and is steadily increasing [6]. Southeast Asia has the highest prevalence of adult myopia (32.9%) of any World Health Organisation (WHO) sub-region [7]. It is estimated that half the world’s population will have myopia by 2050 [8–10]. Uncorrected myopia among young adults in Vietnam, the age group at greatest risk for road traffic death, accounts for 93% of all vision impairment [11, 12]. Although myopia can be safely, effectively and inexpensively treated with spectacles, rates of spectacle ownership among young people who need them are as low as 15–20% in low- and middle-income countries (LMICs) [13, 14].

Good vision is widely perceived to be essential for safe driving, as reflected in motor vehicle licensure laws that require distance vision of a certain standard before receiving a driver’s licence in many countries. However, there is little evidence from existing interventional studies that improving vision enhances traffic safety. Systematic reviews based primarily on studies from high-income countries have largely failed to detect a strong association between central vision and crash risk [15]. However, the situation appears to be different in LMICs. A recent meta-analysis by Piyasena et al. of 13 studies from LMICs revealed a 46% increased risk of road traffic crashes among drivers with impaired central visual acuity (risk ratio [RR] 1.46, 95% confidence interval (CI) 1.20 to 1.78, *p* < 0.001) and a high prevalence of poor vision among drivers in such settings [16]. Although some studies have investigated the impact of vision correction on driving performance [17–19], the review by Piyasena et al. found no randomised trials of vision and driving safety in LMICs [16].

The STABLE (Slashing Two-wheeled Accidents by Leveraging Eyecare) randomised trial was designed in collaboration with the Vietnamese Ministry of Transportation to address these evidence gaps by assessing the hypothesis that the provision of spectacles to young myopic motorcycles drivers in Vietnam can reduce the rate of crash and near-crash (CNC) events, as measured by on-board Data Acquisition Systems (DAS) under conditions of naturalistic driving.

## Materials and methods

STABLE is an investigator-masked, parallel-group, naturalistic driving study using a superiority, stepped-wedge, cluster randomised trial (SW-CRT) design, with faculties within participating universities as the clusters.

### Study design

STABLE was designed as a SW-CRT because of the ethical concerns inherent in failing to deliver spectacles immediately to persons identified with uncorrected myopia and engaged in a potentially dangerous activity such as driving a motorcycle, as would occur in a traditional trial. The stepped-wedge design involves the sequential transition of clusters from control to intervention conditions in randomised order at 12-week intervals, with spectacles delivered to those needing them as soon as uncorrected myopia is detected. After all participant clusters have undergone vision testing and refraction, all eligible participants will have been exposed to the intervention. This approach balances the ethical requirement to avoid withholding treatment from known myopic drivers and the need to conduct a randomised evaluation of the intervention.

### Participants and recruitment procedure

The research team will formally engage with the Board of Executives of selected universities in Ho Chi Minh City, explaining the study’s objectives and extending invitations for their participation. The study will be advertised via posters, email and the fan pages of either the Department of Student Affairs or the Student Union at each enrolled university. To encourage recruitment, advertisements for STABLE will include a brief description of the incentives for participation (VND 220,000 or approximately US$9 per student per month for the duration of involvement in the study) and free spectacles for participants identified with uncorrected myopia (see below).

Prior to recruitment, potential student participants will be asked to conduct a smartphone-based vision self-assessment using WHOeyes, the WHO’s free smartphone application for vision monitoring available in Vietnamese [20, 21]. Those failing that assessment (an estimated 40% based on pilot testing who have distance visual acuity in either eye < 6/12, with normal near visual acuity in both eyes) are at increased risk of refractive error and will be invited to join the pre-evaluation phase of the study. Students providing informed consent and meeting the initial inclusion criteria below on a brief survey will have DAS devices fitted to their motorcycles. These instruments are equipped with front and rear, driver-facing video cameras and accelerometers, and can detect changes in speed and direction (such as swerving).

- Enrolled in one of the selected faculties of a participating university
- Aged 18 to 23 years
- Sole driver of their motorcycle
- Use their motorcycle as their primary means of transport
- Drive ≥ 25 km/week by self-report
- Hold a valid driving licence if required (for motorcycles having an engine capacity > 50 cc)
- Failed the WHOeyes vision self-assessment in at least one eye

**Fig. 1:**
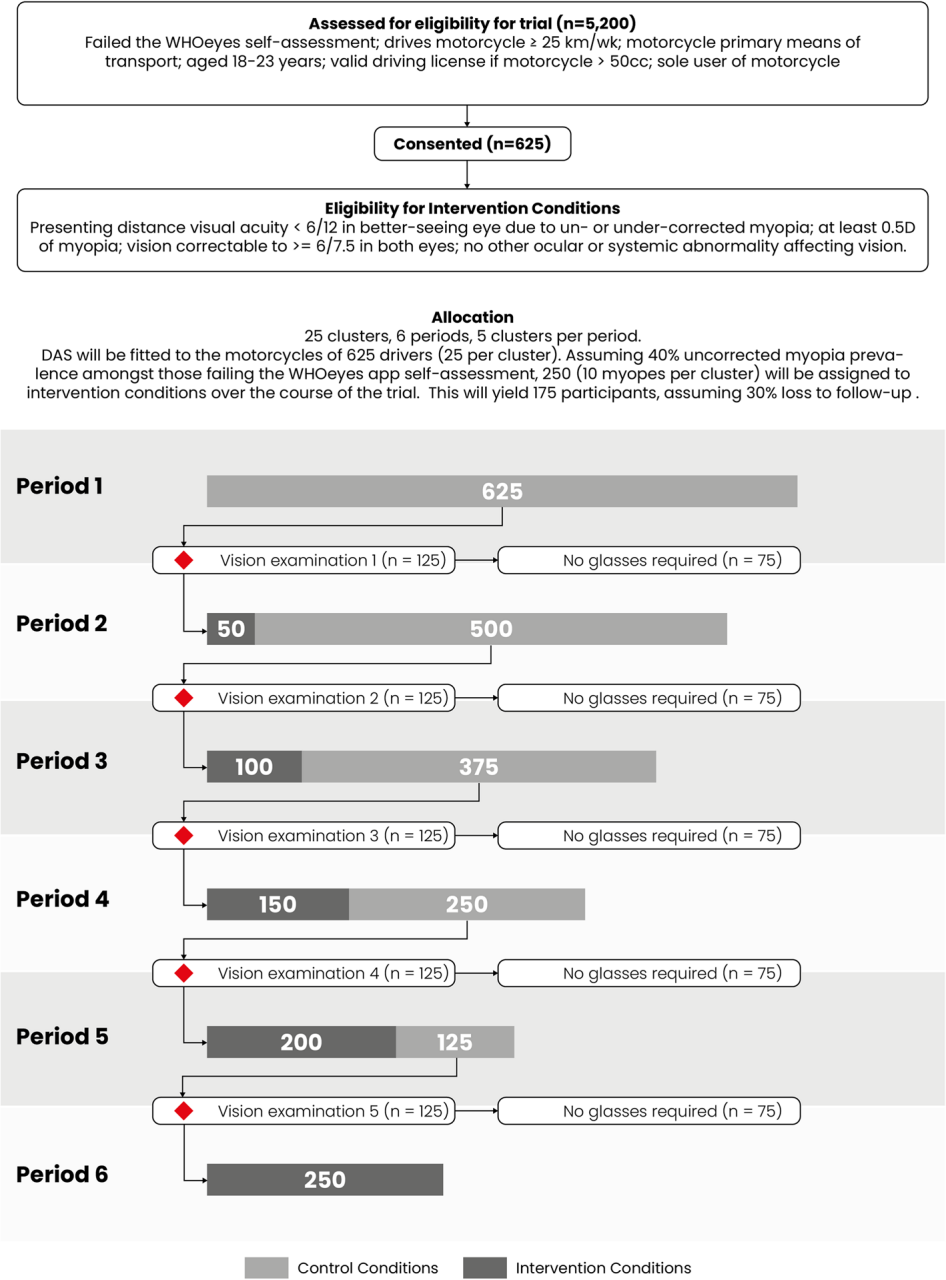
Schematic diagram of participant recruitment and allocation in the STABLE trial

As shown in Fig. 1, all potential participants enter in the control condition and will have DAS units installed on their motorcycles. However, as they have not yet been examined and refracted, the identity of actual trial participants is not known at that time. Subsequently, during the 14 days before each 12-week period, participants in five randomly selected clusters will undergo an eye examination and refraction. Those meeting the following criteria will receive spectacles within 1 week of the examination and transition to the intervention condition, and their previously collected DAS information will be analysed as control data:

- Presenting distance visual acuity < 6/12 in the better-seeing eye due to un- or under-corrected myopia
- Vision correctable with glasses to 6/7.5 or better in both eyes
- At least 0.5 D of myopia in both eyes

Participants who do not meet the above inclusion criteria will have DAS units removed from their motorcycles, never having entered the trial itself, and the data collected previously will not be used in the STABLE analyses.

### Study intervention

Over the 72 weeks of the study, an estimated 250 students from the 25 clusters, randomised in groups of five clusters, will enter the intervention condition. Each of these participants will receive a free pair of spectacles with spherocylindrical power in each eye as measured during refraction by an optometrist. Refractive power will be based on non-cycloplegic automated refraction (QuickSee, Plenoptika, Cambridge, USA) and subjective refinement. Spectacle-wear compliance will be assessed and recorded throughout the trial based on footage from the driver-facing cameras. Replacement spectacles will be provided free of charge as needed due to loss or breakage. Research staff will communicate with trial participants observed having poor compliance in order to improve their use of the spectacles. Myopia progression in this age group is not expected to be clinically meaningful over the 72 weeks, so there are no provisions for routine re-evaluation of refractive power. However, participants complaining of declining vision or symptoms associated with spectacle wear will be examined at study clinics, with replacement spectacles provided as needed.

### Outcome measures

#### Primary outcome

The primary trial outcome is the number of CNC events per 1000 km driven. Study participants will be asked to meet with research staff every 2 weeks to exchange the USB drives from their DAS units for empty drives. Potential CNC events will be extracted from the DAS recordings using mining algorithms in which kinematic data (lateral and longitudinal acceleration due to hard braking events or the vehicle having fallen on its side) will be passed through filters to discover points at which a CNC event may have occurred. Personnel trained in video reduction will then examine the video footage of the candidate CNC event to assess the content, nature and severity.

An analysis of the probability of CNC events will require the collection and analysis of data from periods (epochs) of motorcycle use free from CNC events. The number of baseline epochs collected for each motorcycle driver will be calculated as a percentage of the total number of kilometres travelled by all drivers during the course of the study. The start-point for each baseline epoch will be randomly selected from the drivers’ video data. If the motorcycle is moving more than 5 km/h, 10 s of video data will be extracted and analysed by video reduction personnel who will record roadway (road type and condition) and traffic variables; riding behaviour (overtaking, speed); riding manoeuvres (turning or going straight, etc.); presence of passengers and environmental conditions (e.g. weather conditions) for each baseline epoch interrogated. Assessors will be masked to the participant’s allocation.

#### Secondary outcomes

Secondary outcomes include self-reported visual function (driving-adapted Visual Function Questionnare-25 [VFQ-25]) [22] administered at baseline and 1 month after receiving spectacles; self-reported crashes and injuries reported every 2 weeks by questionnaire (Crash and Injury Self-Report Questionnaire [CI SRQ]); self-reported crashes (compared with DAS-recorded crashes); and total delivery cost per CNC event avoided in the intervention condition (as an indicator of cost-effectiveness).

### Sample size

Unpublished data from Dynamic Vision, an organisation currently collecting CNC events from motorcycle drivers in Vietnam, were used to inform the power calculation. Results showed an estimated mean event rate of 1.67 CNC events per 1000 km driven (standard deviation [SD] = 0.556). For a postulated, clinically meaningful 10% reduction in CNC events, a stepped-wedge cohort design comprising six sequences of seven myopic participants per cluster (a 28% prevalence, below the 40% figure observed during pilot testing with the WHOeyes app), a coefficient of variation of cluster sizes of 3, and five clusters per sequence with Individual Auto Correlation (IAC) of zero, confer 90% power (mean difference = 0.167, SD = 0.556; intraclass correlation [ICC] = 0.02 [0.01–0.05]) [23]. This would be achieved by collecting DAS data from 175 students over the course of 72 weeks. To achieve this, 5200 students will be invited to self-assess their vision using WHOeyes. Pilot data indicate that approximately 12%, or 625 students, will fail that assessment and be eligible to join the preliminary phase of the study, with placement of DAS units on their motorcycles. An estimated 250 (40% of those failing the WHOeyes self-assessment) will have eligible, uncorrected/under-corrected myopia detected on their subsequent eye examination by an optometrist. They will then be assigned to the intervention condition and given free spectacles. With a 30% dropout rate, this will result in complete datasets for 175 participants over the course of the trial.

### Randomisation, allocation and concealment

The random selection of clusters will occur 3 weeks prior to the scheduled date for eye examinations. The identity of the selected five clusters will not be known by the investigators or anyone involved in the trial before randomisation.

The Northern Ireland Clinical Trials Unit (NICTU) will use a centralised system to manage the randomisation process in collaboration with the LV Prasad Eye Institute Clinical Trials Unit (LVPEI CTU). The NICTU will advise LVPEI CTU of the clusters selected 5 weeks before the transition date. Then, LVPEI CTU will inform the site principal investigator or an individual with delegated authority of the five clusters selected. The 125 participants in these randomly selected clusters (25 participants per cluster) will then be notified of the eye examination and reminded 14 days, 5 days and 1 day in advance.

### Reporting of adverse events

The period for reporting adverse events (AE) in the trial commences when informed consent is received from a participant and terminates when the DAS unit is removed from his/her motorcycle. AEs will be recorded and reported in compliance with Vietnamese national guidelines. Any serious adverse events (SAEs) will be promptly reported using the designated form. The LVPEI CTU will be notified of all SAEs by email within 24 h and will notify the chief investigator and sponsor of all SAEs within an additional 24 h. The LVPEI CTU will notify the Queen’s University Belfast (QUB) Research Ethics Committee of all SAEs within 7 days. The local principal investigator will provide annual progress reports to the local IRB.

### Additional data collection

The following information will be collected by questionnaire from those who fail the WHOeyes self-assessment in at least one eye: age, driving experience, primary means of transport, distance driven per week, motorcycle type, brand, model and engine capacity, and possession of a driving licence. Those meeting the inclusion criteria for DAS fitting and providing informed consent will be asked to provide the following baseline data: exposure to eyecare assessments at time of licensure, visual function (VFQ-25) [22], driver risk profile (Dula Dangerous Driving Index [DDDI]) [24] and economic data including home location (urban or rural), income and estimated monthly expenditures. At the time of the eye examination but before the participant’s cluster transitions to the intervention condition, data will be collected on visual acuity, refractive error, details of current spectacle wear, presence of ocular abnormalities and whether spectacles are required. Data will subsequently be collected on whether spectacles are provided.

Every 2 weeks, participants meeting with study staff to exchange the removable drive in their DAS units will be asked whether they have been involved in a crash in the preceding 2 weeks. Repair and insurance costs will be recorded if a crash occurs. Financial data for the cost-effectiveness calculations from a societal perspective will be captured, including cost of health care received and for crash-related repairs.

### Data management

Data from each participant in the preliminary study phase and/or the trial will be recorded in the study database by Asia Injury Prevention Foundation (AIPF) staff. Standard validation procedures will be implemented to ensure consistency, reliability and full compliance with Good Clinical Practice (GCP) [25] and other pertinent regulatory requirements. The database with CNC and compliance information will be under the custodianship of University of Medicine and Pharmacy (UMP). NICTU’s standard procedures for data backup, storage and security will be strictly followed for data entered into the trial management software. Access to these data will be restricted to authorised personnel.

### Statistical analyses

All outcomes for trial participants will be analysed in accordance with the intention-to-treat (ITT) principle. Baseline characteristics will be summarised using means and standard deviations, medians and interquartile ranges, or numbers and percentages, as appropriate. The primary outcome (number of CNC events per 1000 km driven) will be analysed using generalised linear mixed models, reporting mean differences and adjusting for cluster, secular and time effects. For example, secular trend adjustments will take into consideration national and regional driving safety campaigns and calendar time. Seasonal factors (amount of daylight, inclement weather, etc.) are likely to play a role in the number and severity of CNC events. Analysis of secondary outcomes will be done in a similar fashion. Risk differences for binary outcomes and mean differences for continuous outcomes, all adjusting for cluster, secular and time effects, will be reported.

Sensitivity analyses will be conducted to examine the intervention effects, adjusting for additional pre-specified potential confounders including age (years as a continuous variable), DDDI risk profile [24] (total score as a continuous variable) and residence (rural/urban as a binary variable).

Missing data will likely result when participants withdraw from the study or do not complete the full follow-up. Multiple imputations of missing data when assessing secondary outcomes will involve creating 20 copies of the data and imputing missing values by chained equations before averaging the datasets [26].

### Trial monitoring

The trial will be independently monitored by a Data Monitoring and Ethics Committee (DMEC), with the remit to protect and serve study participants, especially regarding safety, and to assist and advise the chief investigator to protect the validity and credibility of the trial. No interim analyses of the trial outcomes are planned.

### Protocol compliance

A protocol deviation is defined as a failure to adhere to the protocol, such as delivering an incorrect intervention or errors in applying eligibility criteria. Major protocol deviations constitute instances where enrolled participants have not provided informed consent or deliberately provide incorrect data. Other protocol deviations include situations where participants are incorrectly randomised, or where participants in the intervention condition have not receive the prescribed spectacles.

### Participant confidentiality

The chief investigator and local principal investigator are responsible for assuring participant privacy. All participant information and records in the study will be pseudo-anonymised with an identification code. Electronic and paper-based data that could reveal a participant’s identity will be securely stored on a server with access limited to designated study personnel, or in locked filing cabinets at UMP until data collection is completed. Anonymised data will be retained for 10 years in accordance with the funder’s requirements (https://www.ukri.org/wp-content/uploads/2020/10/UKRI-020920-ConcordatonOpenResearchData.pdf) and then treated as confidential waste and destroyed.

## Discussion

Road traffic crashes are the leading cause of mortality among people aged 5–29 years, and 90% of this burden falls on LMICs [1]. Poor vision due to uncorrected refractive error in Vietnam and Southeast Asia, especially among young people [7, 11, 12], has been shown in a recent meta-analysis to increase the risk of road crashes by 46% [15]. Despite evidence of the link between good vision and road safety, there are few studies on interventions to reduce crash risk by treating vision disorders such as myopia [17–19]. The recent meta-analysis and review by Piyasena et al. found no randomised trials on vision care and traffic safety in LMICs [16]. The STABLE trial will help to fill this knowledge gap by examining the effects of spectacles to correct myopia to reduce crashes among university students in Vietnam. It will be the first randomised trial of vision interventions and driving safety in a LMIC and a successful trial outcome will help to inform strategies to reduce traffic crashes in the future through wide dissemination and engagement with policy makers.

### Trial status

This paper is based on version 4.2 of the STABLE protocol (dated 15 May 2024). Important changes to the protocol will be communicated to the relevant ethics committees and trial registries as needed. Recruitment to STABLE is expected to start and be completed (given the stepped-wedge design which requires all participants to be included at the outset) in the fourth quarter of 2024.

## Data Availability

Following publication of the results for the primary and secondary outcomes, there may be scope for supplementary analyses by third parties on the data collected. In such cases, written formal requests to access the data must be sent to the chief investigator through the NICTU for discussion with the sponsor. The study will comply with good practice principles for sharing individual participant data from publicly funded clinical trials [27, 28]. Data sharing will be undertaken in accordance with the required regulatory requirements. In the event of publications arising from these analyses, those responsible will provide the chief investigator with a copy of the intended manuscript for approval before submission and acknowledge the source of the data they use. No identifying images or other personal or clinical details of participants are presented here or will be presented in reports of the trial results. The participant information materials and informed consent form are available from the corresponding author on request.

## Abbreviations

LMICs: Low- and middle-income countries
STABLE: Slashing Two-wheeled Accidents by Leveraging Eyecare
DAS: Data Acquisition Systems
CNC: Crash-near crash
WHO: World Health Organisation
SW-CRT: Stepped-wedge, cluster randomised trial
VFQ-25: Visual Function Questionnare-25
CI SRQ: Crash and Injury Self-Report Questionnaire
IAC: Individual Auto Correlation
ICC: Intraclass correlation
QUB: Queen’s University Belfast
NICTU: Northern Ireland Clinical Trials Unit
LVPEI CTU: LV Prasad Eye Institute Clinical Trials Unit
AE: Adverse events
SAEs: Serious adverse events
AIPF: Asia Injury Prevention Foundation
GCP: Good Clinical Practice
DDDI: Dula Dangerous Driving Index
ITT: Intention-to-treat
UMP: University of Medicine and Pharmacy
DMEC: Data Monitoring and Ethics Committee
ICH: International Conference on Harmonisation
ENGINE: Eyecare Nurtures Good-health, Innovation, driviNg-safety and Education

## References

[1] World Health Organization. Global status report on road safety 2015. Geneva: World Health Organization, 2015. Available at https://www.who.int/publications/i/item/9789241565066. Accessed 8 July 2024.

[2] United Nations General Assembly. Transforming our world: the 2030 Agenda for Sustainable Development. 2015. Available at https://www.unfpa.org/resources/transforming-our-world-2030-agenda-sustainable-development. Accessed 8 July 2024.

[3] World Health Organization. Global status report on road safety 2018. Geneva: World Health Organization, 2018. Available at https://www.who.int/publications/i/item/9789241565684. Accessed 8 July 2024.

[4] Peden M, Oyegbite K, Ozanne-Smith J, et al. World report on child injury prevention. Geneva. 2008. Available at https://www.who.int/publications/i/item/9789241563574. Accessed 8 July 2024. 26269872

[5] United Nations General Assembly. Road safety performance review—Vietnam. New York: United Nation; 2018. Available at https://unece.org/DAM/trans/roadsafe/unda/RSPR_Viet_Nam_FULL_e.pdf. Accessed 8 July 2024.

[6] GBD 2019 Blindness and Vision Impairment Collaborators; Vision Loss Expert Group of the Global Burden of Disease Study. Causes of blindness and vision impairment in 2020 and trends over 30 years, and prevalence of avoidable blindness in relation to VISION 2020: the Right to Sight: an analysis for the Global Burden of Disease Study. Lancet Glob Health 2021;9(2):144-160. 10.1016/S2214-109X(20)30489-7 PMC7820391 33275949

[7] Hashemi H, Fotouhi A, Yekta A, Pakzad R, Ostadimoghaddam H, Khabazkhoob M. Global and regional estimates of prevalence of refractive errors: systematic review and meta-analysis. J Curr Ophthalmol. 2018;30(1):3–22. 29564404 10.1016/j.joco.2017.08.009 PMC5859285

[8] Dolgin E. The myopia boom. Nature. 2015;519(7543):276–8. 25788077 10.1038/519276a

[9] Morgan IG, French AN, Ashby RS, et al. The epidemics of myopia: aetiology and prevention. Prog Retin Eye Res. 2018;62:134–49. 28951126 10.1016/j.preteyeres.2017.09.004

[10] Holden BA, Fricke TR, Wilson DA, et al. Global prevalence of myopia and high myopia and temporal trends from 2000 through 2050. Ophthalmology. 2016;123(5):1036–42. 26875007 10.1016/j.ophtha.2016.01.006

[11] Paudel P, Ramson P, Naduvilath T, et al. Prevalence of vision impairment and refractive error in school children in Ba Ria - Vung Tau province. Vietnam Clin Exp Ophthalmol. 2014;42(3):217–26. 24299145 10.1111/ceo.12273 PMC4291105

[12] Limburg H, Gilbert C, Hon DN, Dung NC, Hoang TH. Prevalence and causes of blindness in children in Vietnam. Ophthalmology. 2012;119(2):355–61. 22035577 10.1016/j.ophtha.2011.07.037

[13] Ma X, Zhou Z, Yi H, et al. Effect of providing free glasses on children’s educational outcomes in China: cluster randomized controlled trial. BMJ. 2014;349:g5740. 25249453 10.1136/bmj.g5740 PMC4172821

[14] Wang X, Yi H, Lu L, et al. Population prevalence of need for spectacles and spectacle ownership among urban migrant children in Eastern China. JAMA Ophthalmol. 2015;133(12):1399–406. 26426113 10.1001/jamaophthalmol.2015.3513

[15] Wood JM, Black AA, Dingle K, et al. Impact of vision disorders and vision impairment on motor vehicle crash risk and on-road driving performance: a systematic review. Acta Ophthalmol. 2022;100(2):e339–67. 34309227 10.1111/aos.14908

[16] Piyasena P, Olvera-Herrera VO, Chan VF, et al. Vision impairment and traffic safety outcomes in low-income and middle-income countries: a systematic review and meta-analysis. Lancet Glob Health. 2021;9(10):e1411–22. 34411516 10.1016/S2214-109X(21)00303-X

[17] Nguyen H, Luca Di Tanna G, Coxon K, et al. Associations between vision impairment and driving performance and the effectiveness of visionrelated interventions: a systematic review. Transp Res Interdiscip Perspect. 2023;17:100753.

[18] Chu BS, Wood JM, Collins MJ. The effect of presbyopic vision corrections on nighttime driving performance. Invest Ophthalmol Vis Sci. 2010;51(9):4861–6. 20375338 10.1167/iovs.10-5154

[19] Black AA, Wood JM, Colorado LH, Collins MJ. The impact of uncorrected astigmatism on night driving performance. Ophthalmic Physiol Opt. 2019;39(5):350–7. 31378990 10.1111/opo.12634

[20] World Health Organisation. Check your vision with WHOeyes. Available at https://www.who.int/teams/noncommunicable-diseases/sensory-functions-disability-and-rehabilitation/whoeyes. Accessed 8 July 2024.

[21] Wu Y, Keel S, Carneiro VLA, et al. Real-world application of a smartphone-based visual acuity test (WHOeyes) with automatic distance calibration. Br J Ophthalmol. 2024;108(11):1613–20. 10.1136/bjo-2023-324913. 10.1136/bjo-2023-324913 38514167

[22] Mangione CM, Lee PP, Gutierrez PR, et al. Development of the 25-item National Eye Institute Visual Function Questionnaire. Arch Ophthalmol. 2001;119(7):1050–8. 11448327 10.1001/archopht.119.7.1050

[23] Hemming K, Kasza J, Hooper R, Forbes A, Taljaard M. A tutorial on sample size calculation for multiple-period cluster randomized parallel, cross-over and stepped-wedge trials using the Shiny CRT Calculator. Int J Epidemiol. 2020;49(3):979–95. 32087011 10.1093/ije/dyz237 PMC7394950

[24] Dula CS, Ballard ME. Development and evaluation of a measure of dangerous, aggressive, negative emotional, and risky driving. J Appl Soc Psychol. 2003;33(2):263–82.

[25] International Council for Harmonisation of Technical Requirements for Pharmaceuticals for Human Use (ICH). Integrated addendum to ICH E6(R1): guideline for good clinical practice E6(R2). 2016. Available at https://database.ich.org/sites/default/files/E6_R2_Addendum.pdf. Accessed 8 July 2024.

[26] Azur MJ, Stuart EA, Frangakis C, Leaf PJ. Multiple imputation by chained equations: what is it and how does it work? Int J Methods Psychiatr Res. 2011;20(1):40–9. 21499542 10.1002/mpr.329 PMC3074241

[27] Tudur Smith C, Hopkins C, Sydes MR, et al. How should individual participant data (IPD) from publicly funded clinical trials be shared? BMC Med. 2015;13:298. 26675031 10.1186/s12916-015-0532-z PMC4682216

[28] Tudur Smith C, Nevitt S, Appelbe D, et al. Resource implications of preparing individual participant data from a clinical trial to share with external researchers. Trials. 2017;18(1):319. 28712359 10.1186/s13063-017-2067-4 PMC5512949

[29] World Medical Association. World Medical Association Declaration of Helsinki: ethical principles for medical research involving human subjects. JAMA. 2013;310(20):2191–4. 24141714 10.1001/jama.2013.281053

